# Detection method of small size defects on pipeline weld surface based on improved YOLOv7

**DOI:** 10.1371/journal.pone.0313348

**Published:** 2024-12-12

**Authors:** Xiangqian Xu, Wenting Hou, Xing Li

**Affiliations:** School of Material Science and Engineering, Xi’an Shiyou University, Xi’an, China; Manipal Academy of Higher Education, INDIA

## Abstract

The background of pipeline weld surface defect image is complex, and the defect size is small. Aiming at the small defect size in the weld image, which is easy to cause missed detection and false detection, a lightweight target detection algorithm based on improved YOLOv7 is proposed. Firstly, in the feature fusion network of YOLOv7, the detection ability of the algorithm to detect small and medium-sized targets in defect images is enhanced by adding a 160*160 small target detection head. Then, the convolution module in the backbone network and the feature fusion network is replaced by the depthwise separable convolution with less computational overhead, so as to effectively reduce the network calculation, parameter quantity and model volume. Finally, the loss function CIoU of YOLOv7 is optimized to EIoU loss function to accelerate the convergence speed of the model. The experimental results show that the defect detection mAP@0.5 based on the improved YOLOv7 algorithm can reach 72.2%, which is 11% higher than that of YOLOv7, and the model calculation amount and parameter amount are reduced by 75.6% and 60.3%, respectively. It can completely detect the small size defects and has a high degree of confidence, which can be effectively applied to the detection of small size defects on the surface of pipeline weld.

## 1 Introduction

The welding quality of the pipeline determines the performance and service life of the welded structure. Welding defect detection is an important part of the pipeline before production. During welding, the weldment is affected by the production equipment and process and the experience of the staff, and different degrees and quantities of defects will be formed at the welding site [1]. The generation of these defects is inevitable. If they are ignored, it will affect the performance of the entire pipeline, especially in some application scenarios such as oil field operations. In severe cases, it may bring unpredictable safety accidents.

In order to ensure the quality of welded pipelines, it is necessary to carry out efficient and accurate surface defect detection of welds. However, the traditional manual detection [2] mainly relies on the workers ’ vision, so the reliability of the detection results depends on the experience, professional knowledge and concentration of the inspectors. It has been reported that manual visual inspections of finished products account for 10 percent or more of total labor costs. Further, the accuracy or effectiveness of such visual inspections is only up to 80%, and this level of efficiency is dependent on the implementation of a rigorous and structured inspection process [3].This subjectivity leads to the problems of low efficiency, low detection accuracy and high false detection rate of manual visual defect detection [4]. The pipeline crawling robot can realize the automatic detection of weld surface defects in the pipeline, and achieve high-efficiency detection while reducing labor costs. The vision system of the crawling robot is the key to efficient and accurate identification of weld defects. Therefore, it is of great significance to study a method that can quickly and accurately identify weld surface defects under the background of complex pipeline welds, which is of great significance to improve the efficiency and quality of industrial production.

With the rapid development of artificial intelligence and the continuous improvement of computer computing power, machine learning and deep learning methods have been widely used in the detection of pipeline weld surface defects [5]. Fu et al. [6] proposed a CNN model for acquiring deep-level semantic features of targets, and combined it with multi-receptive fields to realize rapid and accurate classification of steel surface defects. Chen et al. [7] proposed a new Faster R-CNN network model based on the improved ResNet50 to solve the problems of multi-scale target detection environment of weld defect ultrasonic spectrum and poor detection performance of small targets in existing algorithms. Compared with the traditional weld defect detection method, the above method improves the defect detection performance to a certain extent, but there are still problems of low overall detection accuracy and slow detection speed.

The deep learning method can extract the multi-scale features of the target through data set training, improve the accuracy and generalization ability of the model, and is widely used in pipeline weld defect detection [5]. The mainstream target detection networks mainly include: YOLO series [8–13], SSD [14] and Faster RCNN series [15–17]. In terms of pipeline defect detection, Gao et al. [18] proposed an improved YOLOv5 welding defect detection model. By introducing the Rep VGG module to optimize the network structure, the defect detection effect is improved, but it is worth noting that the experimental data were carefully collected in a highly controlled laboratory environment, which naturally reduces the influence of noise sources and unforeseen external disturbances and provides ideal conditions for the initial development and validation of the model. Kou et al. [19] improved the model based on YOLOv3, used Anchor-Free method to improve the speed of the model, and designed dense convolution block to extract richer feature information, thus improving the accuracy and robustness of the model. The improved model achieves 71.3% mAP and 64.5 FPS on GC10-DET and 72.2% mAP and 64.5 FPS on NEU-DET, but the model is too old for the current technology and is not accurate or fast enough to meet the realistic requirements. Although YOLO-z [20] has achieved a good fusion of shallow and middle features by replacing PAFPN with Bi-FPN and expanding the Neck layer, it is not suitable for scenes with large changes in target size. Qian et al. [21] proposed a lightweight defect detection model named LFF-YOLO. By using ShuffleNetv2 as a feature extraction network, the number of parameters was reduced, and the adaptive receptive field feature extraction (ARFFE) module was introduced into the model to accelerate the model convergence. Finally, the mAP@0.5 reached 79.23% on NEU-DET data set and 59.78% on GC10-DET data set, but the model was only for steel surface defect detection. Zhi et al. [22] designed a parallel serial multi-scale feature information fusion mechanism and a channel domain attention strategy to solve the problems of high noise and low recognition accuracy of welding defect data sets, and built a deep learning network model based on Faster R-CNN. The recognition accuracy of this method can reach over 90%. However, the average detection time of 9.8s cannot achieve the goal of real-time detection by the pipeline crawling robot. The above method can effectively learn the target features from the training data, which makes up for the shortcomings of traditional machine learning in manually extracting features, and also improves the accuracy and detection time to a certain extent, but there are still some problems to be solved: (1) The defects in the weld image are small in size, large in number and uneven in distribution, which is easy to cause missed detection and false detection; (2) The existing detection algorithms of pipeline weld defects need to be improved in detection efficiency, and the calculation of the model is too high, which does not meet the requirements of lightweight deployment.

In order to solve the above problems, the small target detection is improved based on YOLOv7 network model in this paper, and the lightweight design idea is taken into account in the model design. By using depth separable convolution to reduce model parameters and calculation, adding small-size target detection layer to strengthen the extraction of small target features by network, and optimizing loss function to accelerate the convergence speed of training.

## 2 Pipeline weld surface defect detection method

### 2.1 YOLOv7 network structure

YOLOv7 [13] is a YOLO model with the best inference speed and recognition effect on the MSCOCO dataset. This model takes into account the detection speed and accuracy, and meets the requirements of fast and accurate recognition of crawling robots in complex environments. Therefore, the YOLOv7 is used as the basic detection model and improves it in this paper. YOLOv7 consists of three parts: Backbone network, Neck network and Prediction network. The Backbone part is composed of integrated convolution unit (CBS), E-ELAN architecture and MP-1 module, which realizes the down-sampling of features and generates the semantic information of the target. The E-ELAN module enhances the feature extraction ability of the network by using CBS to connect residuals in different ways. The MP-1 module samples the input feature map twice by using the parallel connection of Maxpooling and CBS, and performs information fusion. The Neck part uses the PAFPN structure [23,24] to fuse the feature maps from Backbone network.

The loss function of YOLOv7 consists of three parts, which are the binary cross entropy confidence loss function L_obj_, the multivariate cross entropy classification function L_cls_ and the bounding box regression loss function L_box_. The confidence and classification loss functions use cross entropy loss, and the boundary regression loss function uses the CIoU [25], which calculates the loss based on the distance between the center points of the prediction box and the real box, the aspect ratio, and the overlap area.

### 2.2 Improved YOLOv7 weld surface defect detection model

#### 2.2.1 Improvement method

**Fig 1:**
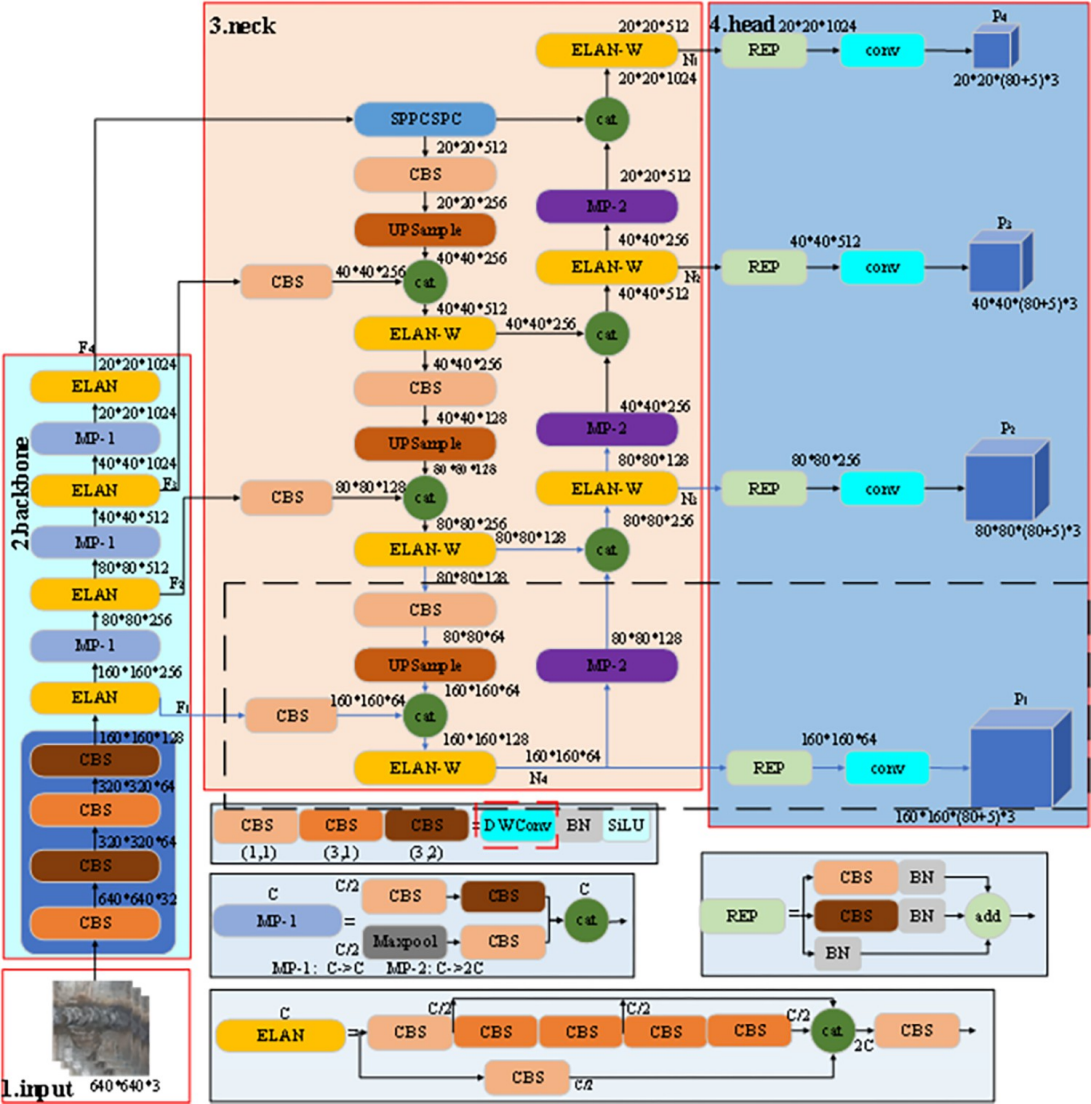
Improved YOLOv7 network structure.

In the task of detecting surface defects in pipeline weld seam images, the traditional YOLOv7 Backbone layer faces challenge in comprehensively extracting features of the defect target due to its small proportion in the image. This difficulty results in a lower final detection accuracy. Therefore, to enhance detection accuracy and efficiency, an improved YOLOv7 model for pipeline weld seam surface defect detection is proposed. In Fig 1, the improved YOLOv7 model architecture shows four core components: input, backbone, neck, and head. The model operates as follows: first, the input is a 640*640*3 image, which is then fed into the backbone network for feature extraction. Based on the three layers of output generated by the backbone, the neck network further fuses and enhances the feature information to generate four different scales of feature maps, which are ultimately used in the HEAD layer to perform the task of target detection and output the final prediction results. For the improved YOLOV7 model, firstly, in the feature fusion part of the network structure, a new detection head dedicated to small target defect detection is added to improve the detection accuracy of small targets. The network structure is circled by the black dotted frame in Fig 1. The input image passes through the backbone network to generate four different scales of feature maps, which are 160*160, 80*80, 40*40, 20*20, respectively. Compared with the original YOLOv7 target detection algorithm, an additional feature map of a size is added. By collecting feature information on more scales, better detection results can be achieved. At the same time, under the idea of lightweight design, the convolution operation in the network structure is simplified, and the depthwise separable convolution with less computation is used, which greatly simplifies the model and reduces the deployment pressure of the model. Finally, the EIoU loss function is used to replace the CIoU loss function as the bounding box loss function to accelerate the convergence speed of the model training.

#### 2.2.2 Small target detection layer

The feature map generated by the YOLOv7 network is more global as the number of network layers deepens, and the semantic information with stronger expression ability can be obtained, but this also causes some fine-grained feature information of the small target it contains to be easily lost. In order to improve the small target perception ability of the YOLOv7 model, a new detection head is added on the basis of the original three detection heads. The detection head outputs a 160*160 large-scale feature map, such as the head part and the dotted box in Fig 1. Firstly, the feature F_1_ extracted from the Backbone part is fused with the features after three times of upsampling to obtain N_4_, and then the detection head P_1_ is obtained after the REP convolution module, which is used to detect small targets. The P_2_, P_3_, and P_4_ detection heads in Fig 1 are all detection heads used to detect small, medium, and large targets in the original YOLOv7 network.

**Fig 2:**
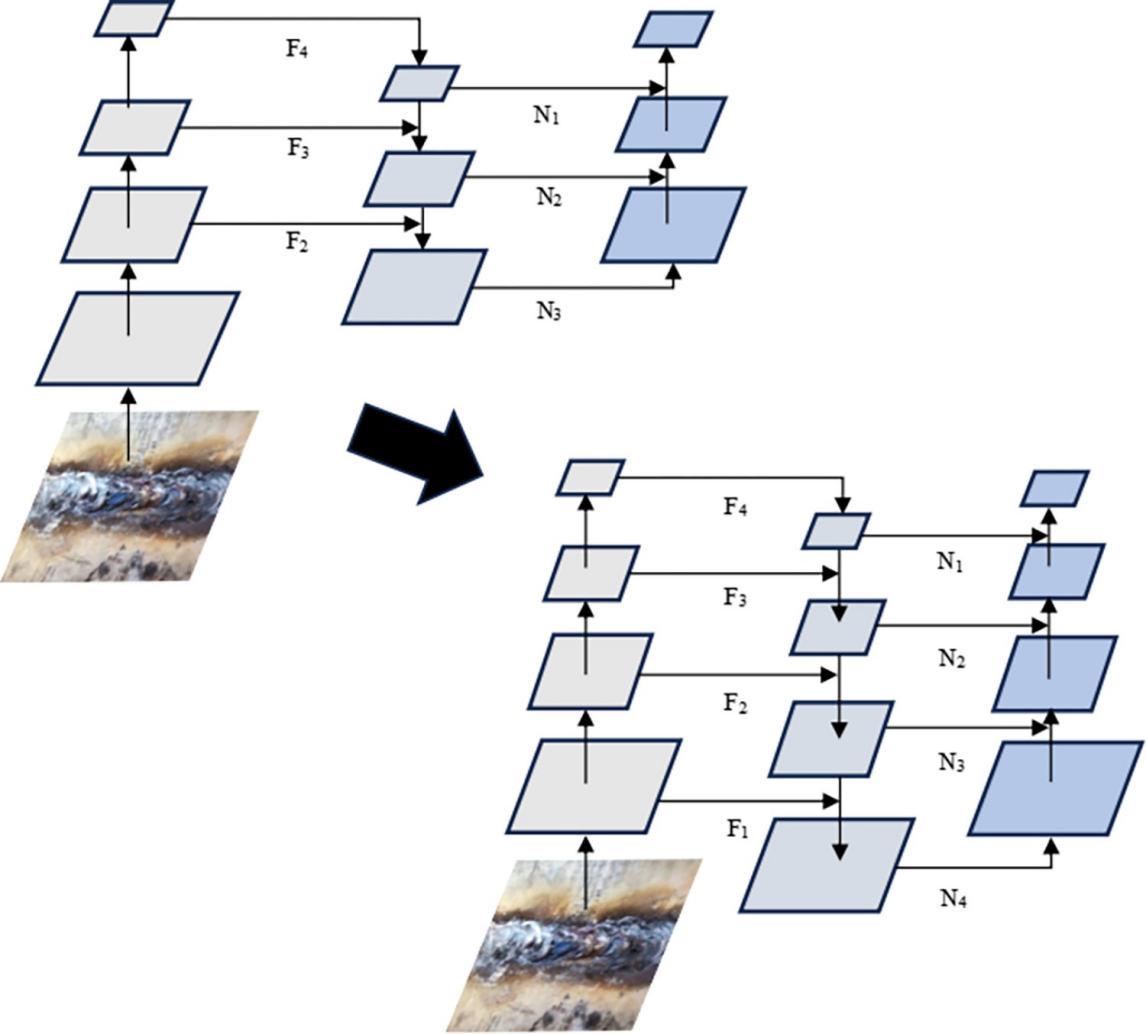
Schematic diagram of feature fusion of small-size target detection head.

By adding the fourth detection branch in the Neck layer, the information fusion of the underlying information features in the shallow network and the high semantic features of the deep network can enable the network to obtain more feature information of small target defects and strengthen the perception of small target defects, thereby improving the detection accuracy of the model for small target defects in complex pipeline weld defect images. The improved network structure is shown in Fig 1, and the schematic diagram of adding the 4th detection branch is shown in Fig 2.

#### 2.2.3 Network lightweight

The network parameters of YOLOv7 amount to 37212738, and its calculation amount reaches 105.2 GFLOPS. However, meeting such high computational requirements is challenging, especially for embedded devices, notably for pipeline inspection robots. Therefore, the YOLOv7 pipeline defect detection model for small targets is restructured using the lightweight MobileNet [26], Employing depthwise separable convolutions from the MobileNet architecture in lieu of regular convolutions significantly reduces both the parameter count and computational load of the network model. As a result, the parameter count of pipeline defect detection model in YOLOv7 is reduced to 14763950, and the calculation amount is reduced to 25.7GFLOPS. As depicted within the red dotted box in Fig 1, the conventional convolutional operations in the CBS module are replaced with DWConv operations.

**Fig 3:**
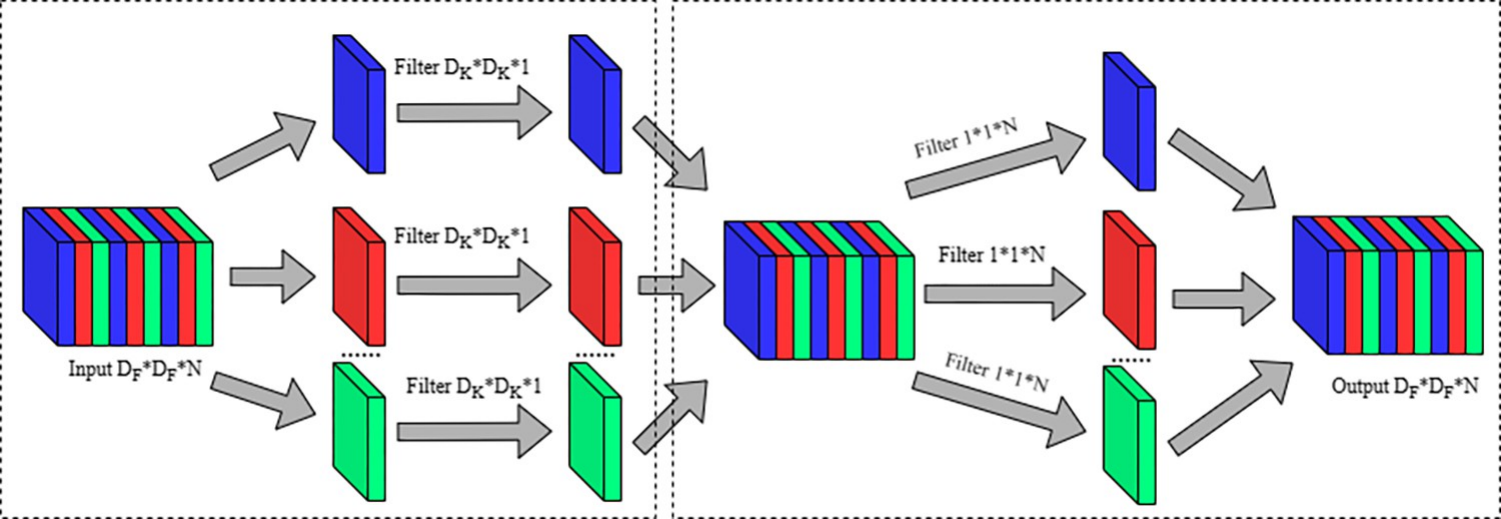
Deepwise separable convolution structure diagram.

The reason why depthwise separable convolution differs from standard convolution and can reduce both parameter count and computational load lies in the fact that standard convolution uses the same convolutional kernel across all input channels. In contrast, depthwise separable convolution operates on each input channel individually, employing distinct convolutional kernels for each channel. It decomposes standard convolution into two operations: depthwise convolution and pointwise convolution. The depthwise convolution utilizes filters with a channel size of 1 to independently convolve each input channel. Throughout this process, Padding and Stride are both set to 1 to ensure that the output feature map maintains the same dimensions and channels. In the process of pointwise convolution, the feature map output by deepwise convolution is convoluted by a filter with a size of 1*1, and the final output feature map is obtained. The size and number of channel of the feature map are consistent with the results of ordinary convolution output. The structure of the deepwise separable convolution is shown in Fig 3.

**Fig 4:**
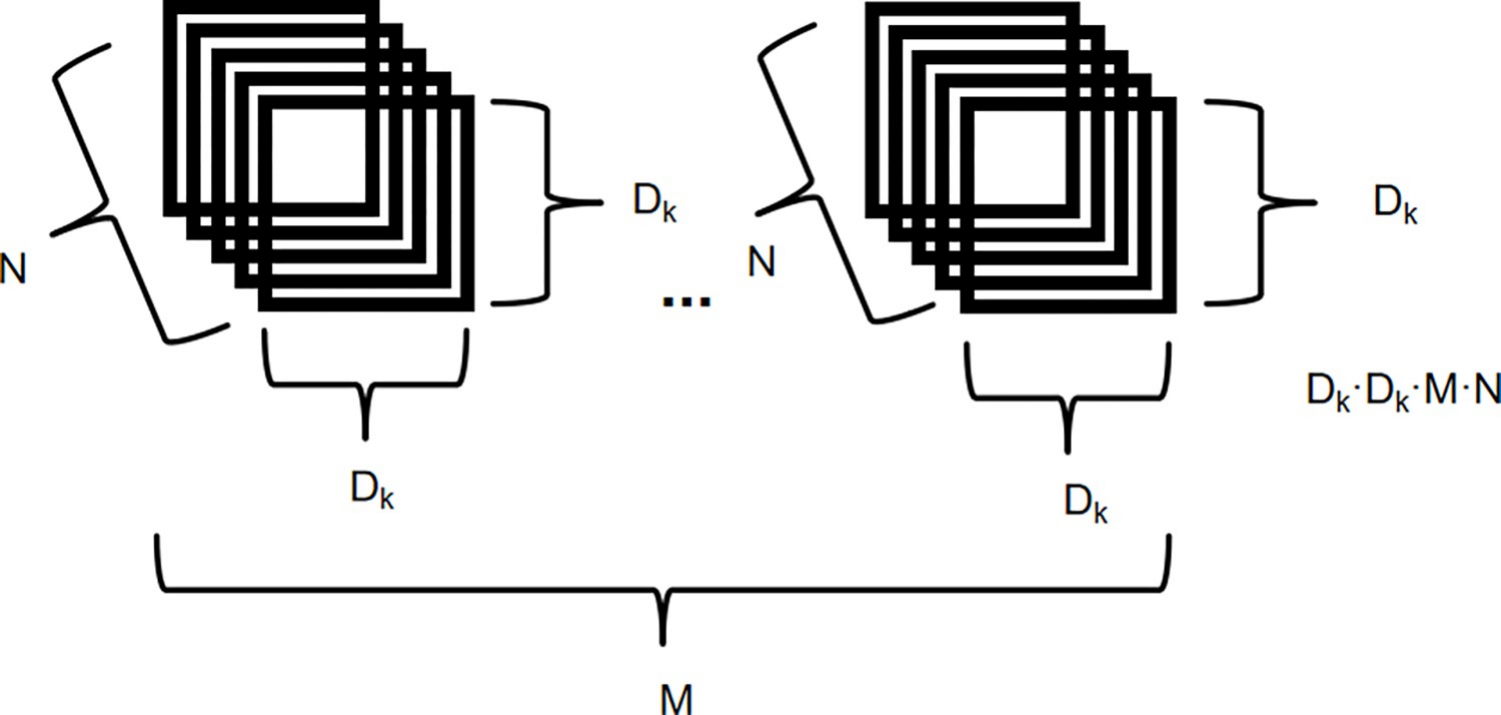
The calculation amount of the ordinary convolution.

Assuming that the ordinary convolution uses M convolution kernels of D_K_ size, the number of channels of the input feature map is N, and the size is D_F_, as shown in Fig 4, the calculation amount of the ordinary convolution is:

$$D_{F}\times D_{F}\times N\times M\times D_{K}\times D_{K}$$ (1)

**Fig 5:**
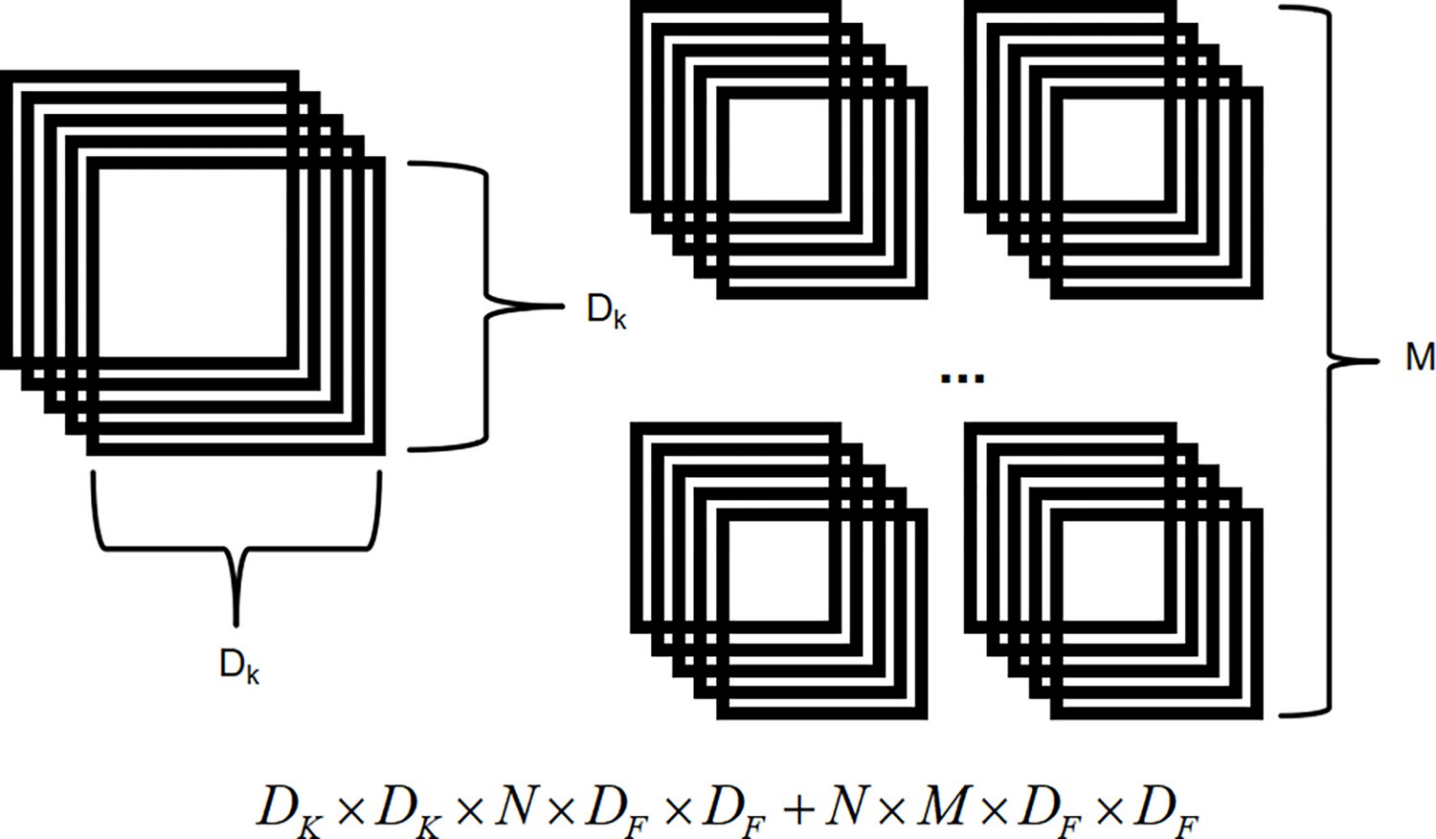
The calculation amount of depthwise Separable Convolution.

Under the premise of using the deepwise separable convolution to replace the ordinary convolution, the calculation is divided into two steps, namely deepwise convolution and pointwise convolution. Depthwise separable convolution is consistent with ordinary convolution in the number of convolution operations, but deepwise convolution is performed first, where each input channel is convoluted independently with a convolution kernel, and then uses pointwise convolution, that is, convolution kernels of size 1x1 and equipped with a plurality of convolution kernels (set to M) are used to combine and generate the characteristics of multiple output channels, as shown in Fig 5. The amount of calculation is:

$$D_{K}\times D_{K}\times N\times D_{F}\times D_{F}+N\times M\times D_{F}\times D_{F}$$ (2)

The ratio of the parameter count of the deepwise separable convolution to the standard convolution is:

$$\frac{D_{K}\times D_{K}\times N\times D_{F}\times D_{F}+N\times M\times D_{F}\times D_{F}}{D_{F}\times D_{F}\times N\times M\times D_{K}\times D_{K}}=\frac{1}{M}+\frac{1}{D_{K}^{2}}$$ (3)

Because in the MobileNet network, the size of the convolution kernel D_K_ is generally 3, which can be deduced from Formula (3). The calculation amount of the depth separable module is 1/9~1/8 of the calculation amount of the ordinary convolution module.

Depthwise separable convolution significantly reduces the number of parameters required, and it processes the data of each input channel independently through the deepwise convolution layer. Thus, the spatial feature information of the original data is effectively preserved. Then, the pointwise convolution layer is used to combine these spatial feature information in an efficient way to generate multiple output channels. This structural design not only reduces the computational complexity, but also maintains a strong ability of feature learning and extraction.

Since the whole network model of YOLOv7 uses standard convolution to complete feature extraction, although it has strong feature extraction ability, it requires strong computational support and storage space. Therefore, the lighter depth separable convolution is used to replace the standard convolution in YOLOv7 model, so as to reduce the parameter count and calculation cost of the network model as much as possible without losing accuracy.

#### 2.2.4 Optimization of loss function

The CIoU loss function used by YOLOv7 considers both the length-width ratio of the regression box and the center distance between the ground truth box and the prediction box. However, when the length-width ratio of the prediction box and the ground truth box is the same, the penalty term for the length-width ratio in the CIoU loss function is always 0, leading to relatively large fluctuations in the convergence process. The calculation formula of CIoU is as follows:

$$loss_{cIoU}=1-IoU+\frac{\rho^{2}(b,b^{bt})}{c^{2}}+\alpha \nu$$ (4)

$$\alpha =\frac{\nu }{(1-IoU)+\nu }$$ (5)

$$\nu =\frac{4}{\pi^{2}}{\left(arctan\frac{w^{gt}}{h^{gt}}-arctan\frac{w}{h}\right)}^{2}$$ (6)

where IoU is the intersection and union ratio of the prediction box and the ground truth box, ranging between [0,1]; b and b^gt^ are the center points of the prediction box and the real box, respectively. *ρ*() represents the Euclidean distance between the center point of the prediction box and the ground truth box, and c is the diagonal distance between the minimum circumscribed rectangle of the prediction box and the ground truth box. *α* is the balance parameter, and *v* is used to measure the proportional consistency between the width and height of the prediction box and the real box.

The penalty terms of the EIoU loss function [27] include aspect ratio loss, overlap loss, and center distance loss. The newly added aspect ratio loss primarily addresses the issue of CIoU not being able to simultaneously increase and decrease the aspect ratio. It directly minimizes the difference in width and height between the ground truth frame and the predicted frame, resulting in faster convergence. To tackle these issues, the EIoU loss function is employed to improve and directly penalize the predictions of width and height, expediting the model’s convergence. The EIoU formula is as follows:

$$loss_{ELoU}=1-IOU+\frac{\rho^{2}(b,b^{gt})}{c^{2}}+\frac{\rho^{2}(w,w^{gt})}{c_{w}^{2}}+\frac{\rho^{2}(h,h^{gt})}{c_{h}^{2}}$$ (7)

where c_w_ and c_h_ are the minimum width and height of the bounding box covering the ground truth box and the prediction box respectively, b and b^gt^ are the center points of the prediction box and the ground truth box respectively, *ρ*() is the Euclidean distance between the center points of the prediction box and the ground truth box, w, h, w^gt^ and h^gt^ are the width and height of the prediction box and the ground truth box respectively.

## 3 Experimental results and analysis

Before carrying out the algorithm model experiment, the data set is first constructed. Subsequently, the network structure and parameters of YOLOv7 algorithm are optimized. Other defect detection algorithms are compared to verify the effectiveness and advancement of the algorithm in the small size defect scene of pipeline weld surface.

### 3.1 Experimental data sets

**Fig 6:**
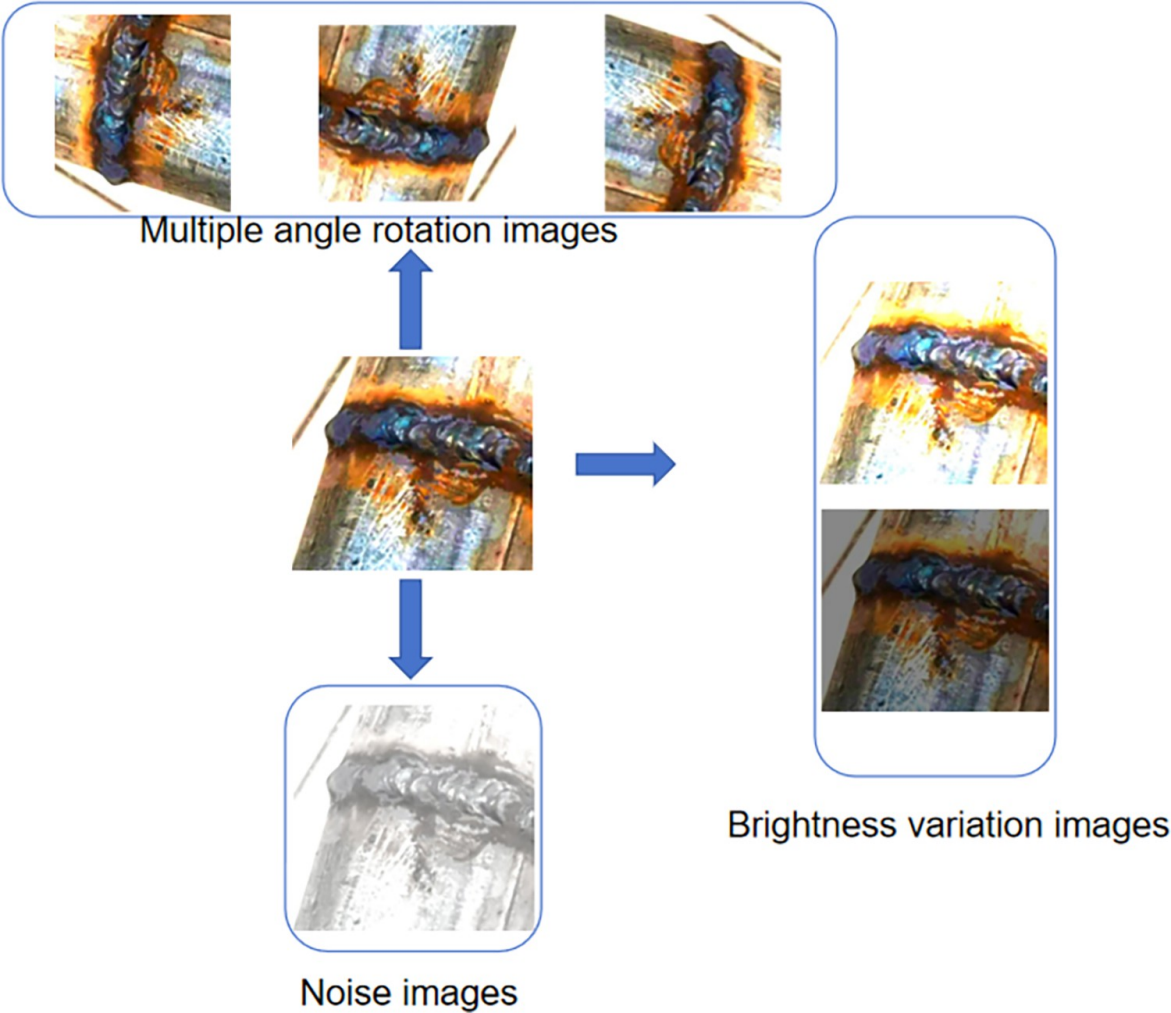
Renderings of data enhancements.

**Fig 7:**
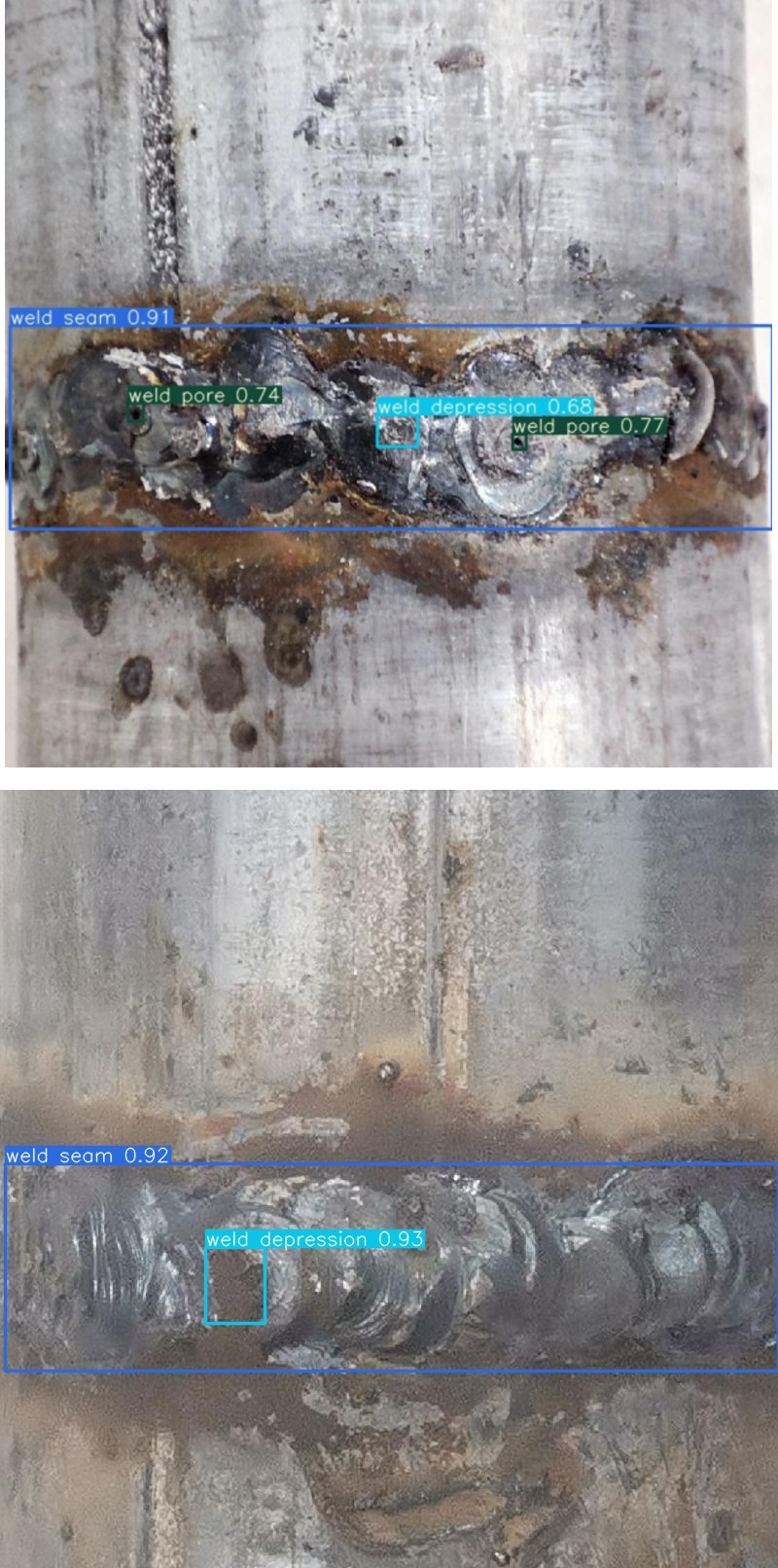
Schematic diagram of weld surface defects.

**Fig 8:**
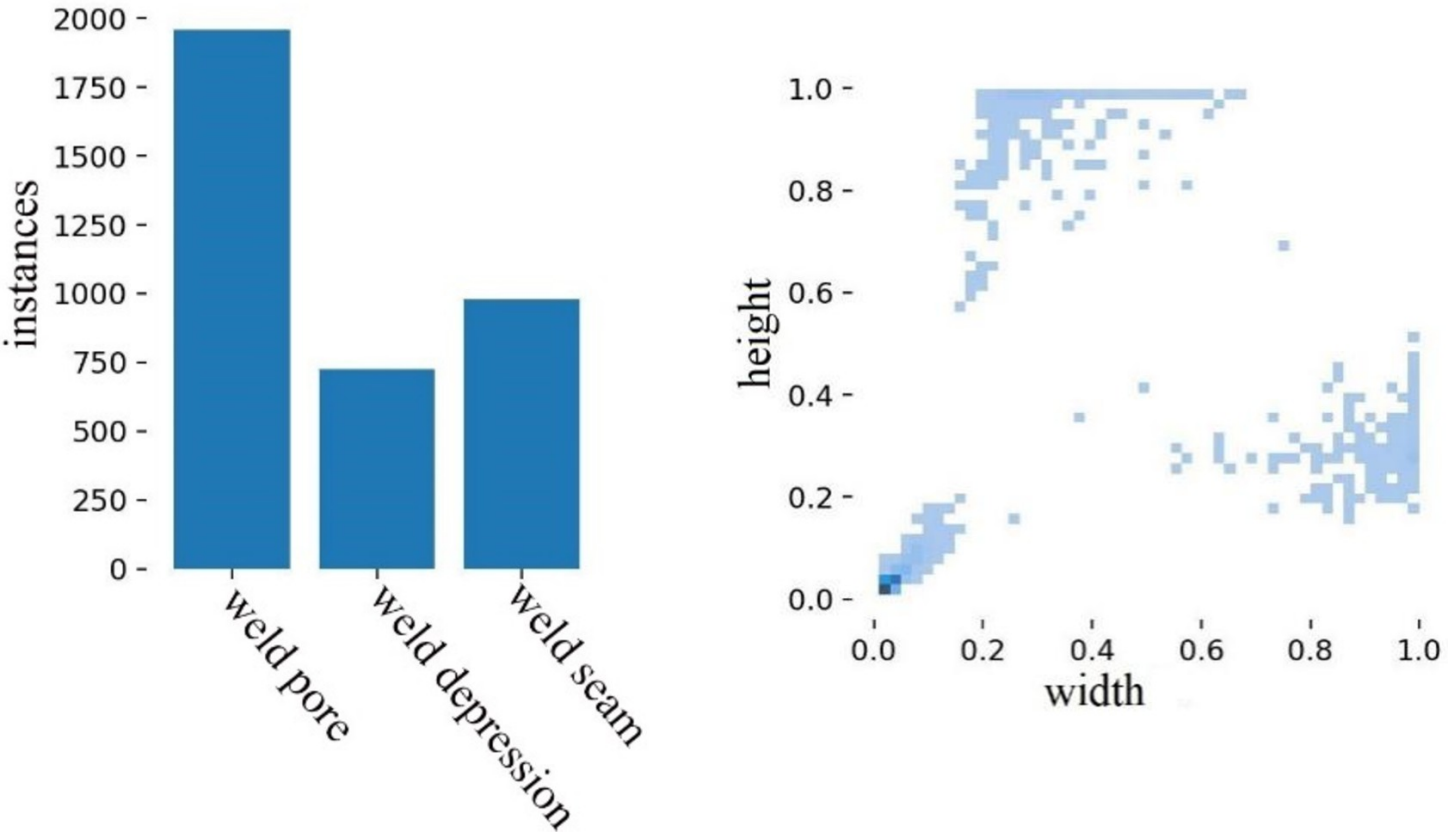
Number and size of marked defects.

The experimentation in this paper draws upon a compilation of image data sourced from both network collection and on-site experimental photography, amassing a repository of over 1500 images. The ratio of defective to non-defective in the entire dataset is 9:1andthe dataset is partitioned into a training set, validation set, and test set, distributed in an 8:1:1 ratio. These images boast a pixel resolution spanning from 800 to 1000. Remarkably, a significant majority of the targets within these images are classified as small targets, delineated by an area smaller than 32*32 pixels. In order to construct a more comprehensive and complete dataset of weld defects, the number of samples is extended using a data enhancement strategy, i.e., morphological operations such as multi-view rotation, saturation adjustment, salt noise, color dithering, etc, are performed on the collected images. It should be noted that in order to avoid data leakage, data enhancement is performed only on the training set after segmentation and the dataset is expanded to 3000 images, as shown in Fig 6. According to the characteristics of the defects in the image, the image defects are divided into weld pore and weld depression, and the weld seam is also included in the detection range. The defect annotation is shown in Fig 7. The size distribution of the marked defects in the image is shown in Fig 8. The left-hand side of Fig 8 shows the statistics of the number of various labels, with the vertical coordinate directly reflecting the number of each label and the horizontal coordinate clearly identifying the specific name of each label. This dataset has sufficient defect sample resources to comprehensively cover and reflect most of the surface defects that may be encountered on pipeline weld surfaces. In Fig 8 right, the horizontal axis is the ratio of the width of the target label to the width of the image, while the vertical axis reflects the ratio of the height of the target label to the height of the image. The dataset covers target objects of various sizes, with small-sized targets occupying a major portion and a wide range of size distributions for these target objects.

### 3.2 Training environment, model parameters and evaluation indexes

The experiment in this paper is completed in a deep learning environment based on Ubuntu 20.04, Python 3.8 and PyTorch 1.10.0. The experiment is carried out on the RTX 4090 server, and CUDA v11.3 is used to accelerate the operation. In the model training, the learning rate is selected as 0.01, the batch size is 16, the number of training rounds is 300, and the input image size is 640*640.

The model identifies and analyzes three types of targets: weld seam, weld pore, and weld depression. Precision, Recall, and mean average precision (mAP) are used as the evaluation indicators of the model’s learning ability in the pipeline weld surface defect target detection task. The volume of the model and the required parameters are used as indicators to measure lightweight.


$$Precision=\frac{TP}{TP+FP}$$ (8)

$$Recall=\frac{TP}{TP+FN}$$ (9)

$$AP=\int_{0}^{1}P(R)$$ (10)

$$mAP=\frac{1}{c}\sum_{j=1}^{c}AP_{j}$$ (11)

In Formula (8) and Formula (9), TP is True Positive, which is a positive example that the test result is true; FN is False Negative, which is a positive case that the detection result is not true; FP is False Positive, which is a negative example that the detection result is true. In Formula (10), AP is the accuracy of a single category. In Formula (11), mAP is the average accuracy of all categories; c is the number of categories.

### 3.3 Experimental results

**Fig 9:**
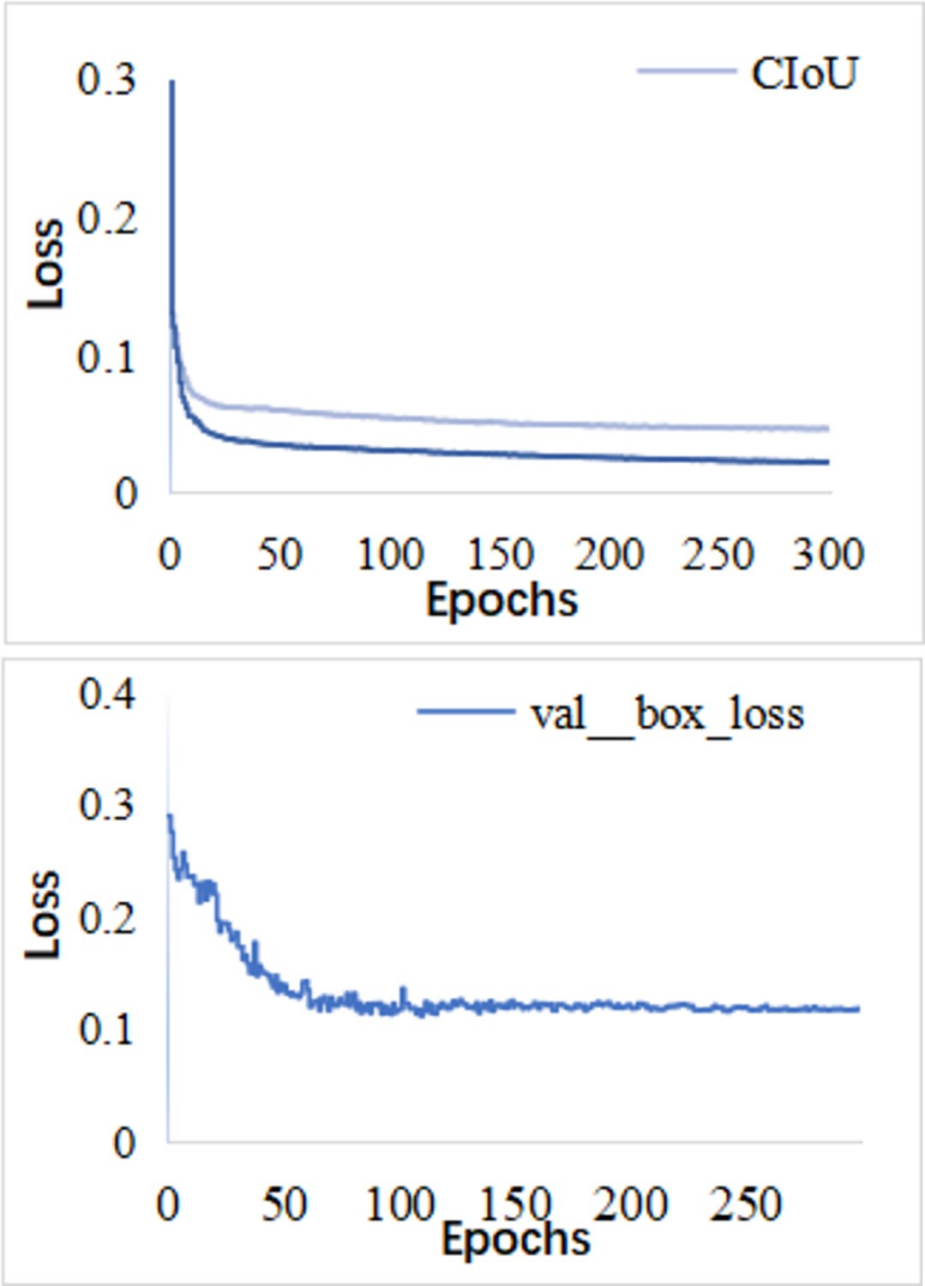
Loss function curve.

The algorithm model in this paper has a total of 499 layers, the total number of parameters is 14763950, about 56.32MB, and the number of floating-point operations performed per second is 25.7GFLOPs. After the training of 300 epochs, the training loss curve obtained is shown in Fig 9. The decline is larger at the beginning of the training, and the downward trend is slower. After 300 rounds of iteration, the overall loss function converges to 0.0422. Also for val box loss it is used to evaluate the model’s localization performance on unseen data. Fig 8 shows that the model converges rapidly before 50 epochs and stabilizes after 150 epochs of iteration. Compared with the original YOLOv7 loss function, the improved loss function after the introduction of EIoU can make the network converge faster. Therefore, the rationality of the improved loss function in this paper is proved.

**Fig 10:**
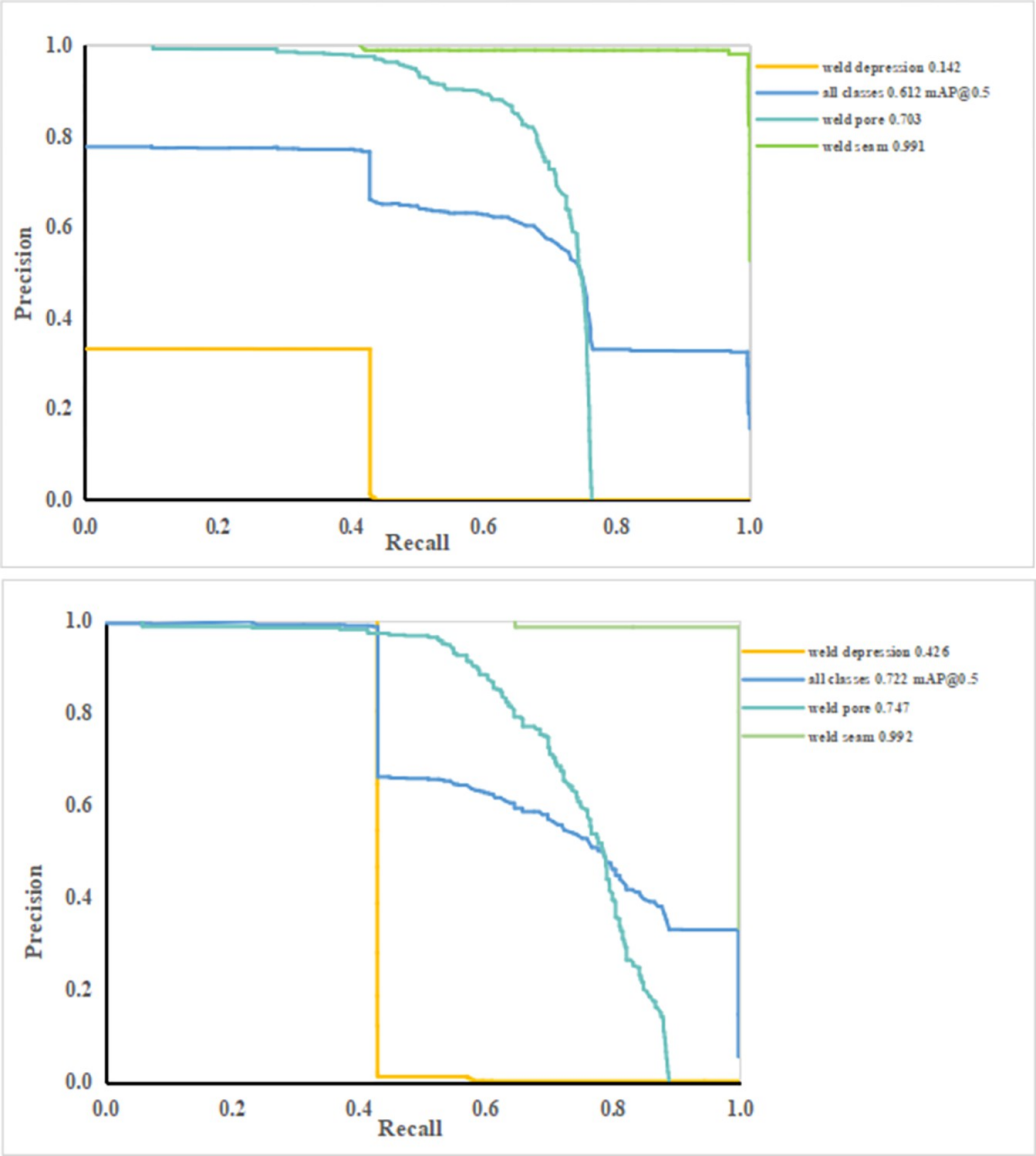
Precision-Recall curve. (a)YOLOv7 (b) Proposed algorithm.

As shown in Fig 10, the Precision-Recall curve of the YOLOv7 algorithm and the proposed algorithm can reflect the overall performance of a model. The abscissa represents the recall rate, and the ordinate represents the accuracy rate. The closer the curve is to the upper right, the better the performance of the model is. It can be seen from the figure that the accuracy of the algorithm in this paper is better than that of YOLOv7 in all categories. The three categories of weld pore, weld depression and weld seam are improved by 0.044, 0.284 and 0.001 respectively. The results show that by introducing small target detection layer, which fuses the underlying features of the shallow network with the high-level semantic information of the deep network, the recognition ability of small target defects is enhanced, which in turn improves the accuracy of the model in detecting small target defects in the defective images of complex pipe welds.

#### 3.3.1 Ablation experiments

In order to verify the effectiveness of the improved points proposed in this paper in the small target defect inspection scenario, the original YOLOv7 is used as baseline, and then the proposed improved strategy is added to the original model one by one, and experiments and tests are carried out. The results of the ablation experiment are shown in Table 1.

**Table 1:** Ablation experiment results.

Experimental serial number	Small size target detection head	Depthwise separable convolution	EIoU	mAP@0.5	GFLOPs
1	×	×	×	61.2%	105.2
2	√	×	×	69.5%	119.5
3	×	√	×	66.9%	21.0
4	×	×	√	61.4%	105.2
5	√	√	×	69.7%	25.7
6	√	×	√	69.6%	119.5
7	×	√	√	67.2%	21.0
8	√	√	√	**72.2%**	**25.7**

It can be seen from the results of the ablation experiment that after adding the small size target detection head, mAP@0.5 has been significantly improved by 8.3%, and the calculation amount has increased slightly by 14.3GFLOPs. As shown in Experiment 2, it can be seen that after adding the small-size target detection head, the detection performance of the model has been improved. In Experiment 3, depthwise separable convolution is introduced on the basis of the original network. Compared with Experiment 1, mAP@0.5 increased slightly, but the calculation amount decreased by 84.2GFLOPs, which proved that the lightweight improvement of the network structure had a significant effect. Experiment 4 introduced the EIoU loss function on the basis of the original model, and mAP@0.5 increased by 0.2% compared with the original model. Experiment 5 also introduced a small-size target detection head and deepwise separable convolution, which greatly reduced the computational complexity of the model on the premise that mAP@0.5 increased. In Experiment 6 and Experiment 7, the above improved strategies are combined into the original model, and mAP@0.5 is increased by 8.4% and 6.0%, respectively. In Experiment 8, the above three improved strategies are all introduced into the original model, and mAP@0.5 also reached the peak, which is 11% higher than the original model, and the calculation amount of the model is reduced by 79.5GFLOPs.

In summary, after the introduction of small size target detection head, deepwise separable convolution, and EIoU, the average accuracy of the model reaches the highest. Each module plays a positive role in the detection of small size target defects on the weld surface, and after the introduction of deepwise separable convolution, the calculation amount of the model has dropped significantly.

#### 3.3.2 Comparative experiments of different detection models

In order to verify the advanced nature of the algorithm proposed in this paper, it is compared with the mainstream classical algorithm. The experimental results are shown in Table 2. Under the premise of inputting the same size image, the proposed algorithm performs best in average detection accuracy compared with other mainstream classical algorithms, which can reach 72.2%, which is 13.5%, 12.7%, 11.1%,9.7% and 15.1% higher than Faster R-CNN, SSD, YOLOv3, YOLOv5-L, and YOLOV8-N respectively, the number of parameters decreased by 5.2%, 71.0%, 76.0% and 29.3%, respectively, the amount of calculation decreased by 24.9%, 50.1%, 83.5% and 46.8% respectively. In addition, compared with YOLOv7 algorithm, the average detection accuracy of the algorithm proposed in this paper increases by 11%, while the parameters and calculation amount are significantly reduced by 60.3% and 75.6% respectively. Although the number of YOLOV8-N parameters and the amount of computation are small, the performance and accuracy of the algorithm proposed in this paper is higher than that of YOLOV8-N in line with practical applications.

**Table 2:** Comparison experiment results.

Algorithm	Input size	Precision	Recall	mAP@0.5	FPS	Parameters (MB)	GFLOPs
Faster R-CNN [28]	640*640	78.2%	64%	58.7%	29	59.4	34.2
SSD512 [29]	640*640	79.1%	69%	59.5%	45	194.5	51.5
YOLOv3 [30]	640*640	75.0%	72%	61.1%	56	234.6	155.3
YOLOv5-L [31]	640*640	82.7%	76%	62.5%	83	79.7	48.3
YOLOv7 [13]	640*640	95.6%	73%	61.2%	91	142.0	105.2
YOLOv8-N [32]	640*640	62.7%	58%	57.1%	140	30.11	8.2
Proposed algorithm	640*640	96.4%	**82%**	**72.2%**	**101**	**56.32**	**25.7**

According to the above data, the improved YOLOv7 algorithm has a significant improvement in the number of model parameters and the amount of calculation, and also has a significant improvement in the average detection accuracy. At the same time, the detection speed can be maintained at about 101 frames per second. It can meet the accuracy and speed requirements of pipeline crawling robot in detecting small targets of pipeline weld surface defects.

### 3.4 Defect detection effect and analysis of the model

**Fig 11:**
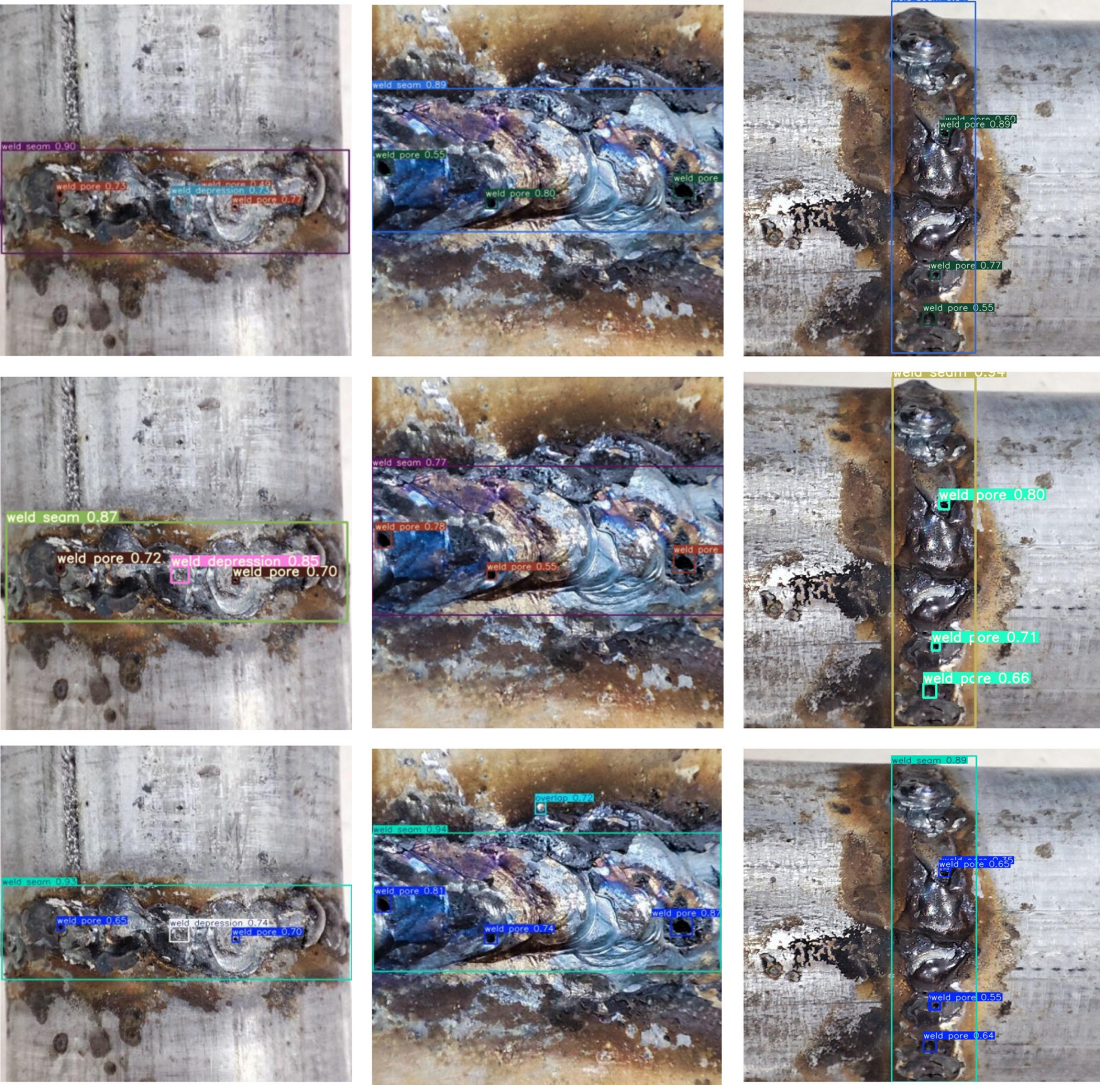
Target detection results (the detection results of proposed algorithm are on the left and YOLOv7 is on the middle YOLOv8-n is on the right).

In order to more intuitively show the detection ability of the algorithm proposed in this paper on the small size defect data set of pipeline weld surface, some small size defect weld images are selected for detection. As shown in Fig 11, all weld defect sizes in the figure are less than 32*32. The three images in the left column of Fig 11 are the detection results using the proposed algorithm, and the three images on the middle side are the detection results using the original YOLOv7 algorithm, the three images on the middle side are the detection results using the original YOLOv8-N algorithm Obviously, for small size defects, the algorithm in this paper can detect them comprehensively and accurately, while the detection results of YOLOv7 and YOLOv8-N algorithm have different degrees of missed detection, and some detection confidence is not as good as the algorithm proposed in this paper. YOLOv 7 and YOLOv 8-N algorithms have leakage in the first row of Fig 11; in the second row of pictures, this paper’s algorithm has a higher confidence in detecting weld porosity defects; in the third row of pictures, YOLOv 7 does not detect all the porosity defects. The Proposed algorithm is more comprehensive in detecting the porosity defects and the algorithm is more reliable in detecting the porosity defects.

It can be seen that after introducing the small-size target detection head into the YOLOv7 network structure, the context information of the defect target is captured with a larger receptive field, so that the network can better learn more small-size target detail features. In the neck layer, pyramid feature fusion is used to further strengthen the network ’s multi-scale defect target detection ability. In addition, the depthwise separable convolution is used to compress the calculation amount of the model to about 1/8 of the calculation amount of the original model, which further improves the training and reasoning speed of the model. Compared with the YOLOv7 algorithm, the proposed algorithm has faster detection speed. It can meet the real-time requirements of pipeline crawling robot detection.

**Fig 12:**
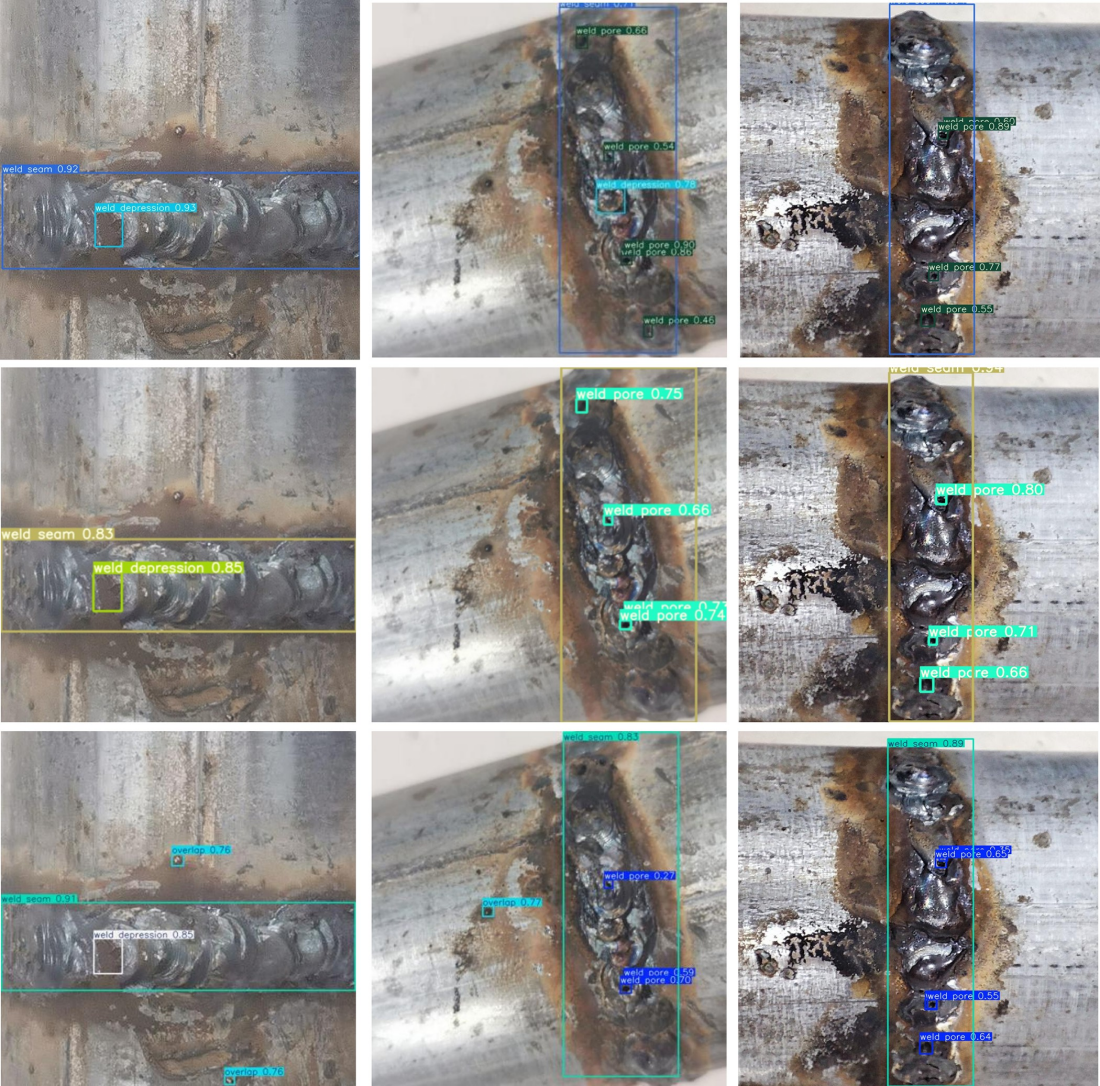
Target detection results in different scenes (the detection results of proposed algorithm are on the left and YOLOv7 is on the middle YOLOv8-n is on the right).

In order to verify the robustness of the proposed algorithm, the weld defects under different light, angle and sharpness are selected as the detection objects. The detection results are shown in Fig 12. Through the first two images in Fig 12, it can be seen that the algorithm in this paper can still detect the defect target with high confidence in the case of weak light. This shows that the improved YOLOv7 algorithm has a certain light adaptability and can adapt to the defect detection task under various light conditions. The weld defect images of the second row and the third behavior at different angles in Fig 12 show that the algorithm proposed in this paper can effectively detect each small size target defect in the image, and can accurately identify their location and category, showing strong robustness and accuracy. In the first, second, and third rows of Fig 12, YOLOV8-N is found to have varying degrees of missed detections while the present algorithm has a higher confidence level. In the weld image of the fourth behavior in the fuzzy shooting case in Fig 12, it can be seen that the YOLOv7 algorithm cannot correctly identify the weld, while the algorithm in this paper can accurately identify the weld.

## 4 Conclusion

Aiming at the small size and large number of surface defects of pipeline welds, which are easy to cause missed detection and false detection, a new lightweight design of small size defect detection algorithm for weld surface, namely improved YOLOv7 algorithm, is proposed.

1. To effectively improve the detection accuracy of small-sized defect targets, a dedicated detection head designed for small targets has been added to the original network. Additionally, ordinary convolutions have been replaced with computationally more efficient depthwise separable convolutions, reducing both the model’s parameter count and computational load. Furthermore, the YOLOv7 algorithm’s CIoU loss function has been optimized to the EIoU loss function, accelerating the convergence of the model.
2. The mAP@0.5 of the improved YOLOv7 algorithm is 72.2%, which is 11% higher than that of the YOLOv7 algorithm. While ensuring the detection accuracy, the calculation amount and parameter count of the model have been significantly reduced by 75.6% and 60.3%, respectively, compared to YOLOv7. Confronted with the task of detecting small-sized defects on the surface of complex pipeline weld seams, the enhanced algorithm demonstrates excellent stability and robustness.
